# HPV positive*,* wild type *TP53*, and p16 overexpression correlate with the absence of residual tumors after chemoradiotherapy in anal squamous cell carcinoma

**DOI:** 10.1186/s12876-018-0758-2

**Published:** 2018-02-21

**Authors:** Paulo C. Soares, Eliana S. Abdelhay, Luiz Claudio S. Thuler, Bruno Moreira Soares, Samia Demachki, Gessica Valéria Rocha Ferro, Paulo P. Assumpção, Leticia Martins Lamarão, Luis Felipe Ribeiro Pinto, Rommel Mario Rodríguez Burbano

**Affiliations:** 1 Hospital Ophir Loyola, Belém, Pará 66060-281 Brazil; 2 Universidade do Estado do Pará, Belém, Pará Brazil; 3 Instituto Nacional de Câncer, Rio de Janeiro, Brazil; 4 Laboratório de Citogenética Humana, Instituto de Ciências Biológicas, Belém, Pará Brazil; 5 Núcleo de Pesquisas em Oncologia, Universidade Federal do Pará, Belém, Pará Brazil; 6 Fundação de Hematologia e Hemoterapia do Pará, Belém, Pará Brazil

**Keywords:** HPV, HIV, p16, *TP53*, Anal squamous cell carcinoma

## Abstract

**Background:**

Anal residual tumors are consensually identified within six months of chemoradiotherapy and represent a persistent lesion that may have prognostic value for overall survival. The aim of this study was to evaluate the association of HPV and HIV status, p16 expression level and *TP53* mutations with the absence of residual tumors (local response) in Squamous Cell Carcinoma (SCC) of the anal canal after chemoradiotherapy.

**Methods:**

We performed a study on 78 patients with SCC of the anal canal who submitted to chemoradiotherapy and were followed for a six-month period to identify the absence or presence of residual tumors. HPV DNA was identified by polymerase chain reaction and direct sequencing, HIV RNA was detected by TaqMan amplification, p16 expression was detected by western blotting, and the mutational analysis of *TP53* was performed by direct sequencing; additionally, samples carrying mutations underwent fluorescent in sit hybridization. The evaluation of the tumor response to treatment was conducted six months after the conclusion of chemoradiotherapy. The following classifications were used to evaluate the outcomes: a) no response (presence of residual tumor) and b) complete response (absence of residual tumor).

**Results:**

The significant variables associated with the absence of residual tumors were HPV positive, p16 overexpressed, wild-type *TP53*, female gender, and stages I and II. Only the presence of HPV was independently correlated with the clinical response; this variable increased the chances of a response within six months by 31-fold.

**Conclusions:**

The presence of HPV in tumor cells was correlated with the absence of a residual tumor. This correlation is valuable and can direct future therapeutic approaches in the anal canal.

## Background

Squamous cell carcinoma (SCC) of the anal canal has an annual incidence in western countries of 1.0 to 2.5 per 100,000 people, with a female predominance of 2:1. It is strongly associated with human papillomavirus (HPV) and human immunodeficiency virus (HIV) infection and is also significant in immunosuppressed patients following solid organ transplantation [1].

Understanding prognostic biomarkers provides information on how biological factors mediate disease progression and response. The integration of this understanding may allow identification of treatments and facilitate a new therapeutic intervention for all patients with SSC. The use of predictive biomarkers of response may also facilitate the modulation of chemoradiotherapy components and the early detection of relapse, which would facilitate rescue surgery. In addition, there is the potential to investigate whether underlying genomic factors modulate the transformation of HPV-induced tumor [2].

Some studies have sought to identify predictive oncobiological factors of local response, disease-free time, and overall survival. Of particular note are the papers that emphasize the role of HPV high-risk genotypes, p16 protein expression and *TP53* gene disruption in oropharyngeal and anogenital SCC [3–11]. These studies demonstrate that patients with positive HPV tumors have a better response to standard therapy, as well as those who over-express p16 and do not have a *TP53* mutation.

There is still no consensus on the best time to define the lack of clinical response to the treatment of carcinomas. Some prospective studies evaluated the response within 4 weeks after completion of chemoradiation, performing biopsies for pathologic determination within 4 to 6 weeks, rather than longer periods for better evaluation of local tumor regression. An 80% regression after chemoradiation may predict colostomy-free survival and disease-free survival. Most randomized studies, however, assess the tumor response between 6 and 12 weeks after chemoradiation. Disparities in response rates in several studies may be partially explained by differences in how and when response to treatment was performed [1]. Results from more recent studies show that the proportion of people with a complete clinical response 6 months after initiation of treatment is more strongly associated with progression and mortality compared to any assessment in less time [12].

The current literature concerning local clinical response after treatment indicates that, when there is a suspicion of a residual tumor, digital rectal examination, anoscopy, biopsy, and less frequent imaging techniques, such as endorectal ultrasound, MRI or PET/CT, should always be performed. This is because the most common recurrence sites are near the primary tumour site (75%), compared to metastatic sites such as presacral, iliac (21%) and inguinal (4%) lymph nodes [13, 14].

Even though PET/CT is not a commonly used imaging exam (in the Brazilian public health system) to observe how anal tumors behave, it has been considered an alternative method to evaluate clinical response and disease relapse [15]. A full metabolic response consists of the absence of FDG (2-Deoxy-2-[18F] fluoro-D-glucose) detection in regions where tumors were detected in pre-treatment investigations, while a partial response is defined as an increase or persistence of FDG captations by tumor cells in the same sites. A partial response assessed by post-therapy PET/CT is significantly associated with disease-free survival (22% versus 95%). The elevated negative predictive value (ranging between 94 and 98%) of PET/CT is important and avoids unnecessary biopsies or surgeries in patients with irradiated tissue [13, 16].

To the best of our knowledge, this type of study has not been conducted in South America. Thus, the objective of this study was to identify the presence of various HPV genotypes, as well as HIV and *TP53* mutations, to quantify the p16 expression levels in patients with SCC of the anal canal and to analyze the associations of the variables with local clinical responses to chemoradiotherapy.

## Methods

### Clinical data retrieval

Clinical details were retrieved for 78 patients treated with radical chemoradiotherapy for stages I, II, IIIa and IIIb SCC of the anal canal between 2003 and 2014. The staging was based on the American Joint Committee on Cancer tumor–node–metastasis (TNM) staging system. The treatment used for all patients was the Nigro protocol [17], which consisted of 50.4 Gray of external radiation divided into 28 daily doses and concurrent treatment with 5-flourouracil and mitomycin.

### Study design

- Seventy-eight patients diagnosed with SCC of the anal canal underwent chemoradiation.
- Three months after the end of treatment, all 78 patients made the first evaluation of local response with standard anoscopy, digital rectal examination, and clinical examination of the inguinal region. The objective was to identify if any patient was presenting a lesion progression.
- Six months after the end of treatment, all 78 patients underwent a new evaluation with computer tomography (CT) of the chest, magnetic resonance imaging (MRI) of the abdomen and pelvic, digital rectal examination, high-resolution anoscopy, and clinical examination of the inguinal region. Those who were suspected of a residual lesion underwent a new biopsy to confirm histopathological suspicion of the residual lesion.
- Patients who had residual lesions confirmed by biopsy were referred for surgical treatment.
- The results of the HPV, HIV, *TP53* and p16 analyses of all 78 patients were only known after 6 months of treatment (blind to those who evaluated the response and to those who did the molecular analyses).

### Analysis of the clinical response

The evaluation of the tumor response to treatment was conducted six months after the conclusion of chemoradiotherapy for all patients and consisted of chest CT, abdomen and pelvic MRI, digital rectal exam, high-resolution anoscopy and clinical examination of the inguinal region. The following classifications were used to evaluate the outcomes: a) no response (presence of residual tumor) and b) complete response (absence of residual tumor). Only patients who showed a complete absence of an ulcerative or vegetative lesion were classified as responding. When a suspicious lesion was detected in the anorectal mucosa, biopsy, and histopathology were conducted. All patients followed-up every three and six months.

### Pathology review

Haematoxylin and eosin stained slides of formalin-fixed paraffin-embedded (FFPE) samples were reviewed to confirm the presence of tumors in the blocks. The samples were microdissected by a pathologist and selected for the study.

### DNA extraction

DNA extraction was performed using a QIAGEN (Venlo, Netherlands) QIAamp DNA FFPE Tissue kit.

### HPV analysis by direct sequencing

Samples were submitted to polymerase chain reaction (PCR) using viral gene-specific primers, which amplified a 150 bp fragment of the *L1* gene corresponding to a conserved region of the virus genome. After PCR, virus identification was performed by direct sequencing of the PCR product using a capillary sequencer 3730XL DNA Analyzer (Thermo Fisher Scientific, USA).

### HIV detection

Viral RNA was extracted using a QIAGEN (Venlo, Netherlands) QIAamp Viral RNA Mini Kit and transferred to COBAS TaqMan HIV-1 Test, v2.0 (Roche, Pleasanton, CA) for amplification and detection.

### *TP53* mutation analysis by nucleotide sequencing

The *TP53* gene has 11 exons. Exon 1 is non-coding in the human p53 protein and for this reason was not analyzed in this study. Exons 2–11 of the *TP53* gene were separately amplified by PCR using the specific primer sets described in Faria et al. [18] The direct sequencing analysis of the amplified products was performed using the automatic sequencer ABI Prism 3130 (Thermo Fisher Scientific, USA). The resulting sequences were directly edited on the Sequencing Analysis Software on a computer linked to a 3730XL DNA Analyzer (Thermo Fisher Scientific, USA).

### Fluorescent in situ hybridization

Only tumor samples carrying mutations in the *TP53* gene were analyzed by fluorescent in situ hybridization (FISH). To determine the chromosome 17 and *TP53* copy numbers, the cells were hybridized using a dual-colour direct labelled probe (Abbott/Vysis, USA) specific for the chromosome 17 α-satellite (SpectrumGreen; p11.1-q11.1) and the *TP53* gene region (SpectrumOrange; 17p13.1). Nuclei were counterstained with DAPI/antifade. For each tumor, 200 interphase nuclei were analyzed and scored using the criteria from Hopman et al. [19]

### Western blotting

The analysis was performed as previously described by Leal et al. [20] Reduced protein from each sample was electroblotted onto a polyvinylidene fluoride (PVDF) membrane (GE, USA). The PVDF membrane was blocked and incubated with the primary antibodies: anti-p16 (dilution 1:10; Thermo Fisher Scientific, USA) and anti-ACTB (dilution 1:250; Thermo Fisher Scientific, USA). After washing, a peroxidase-conjugated secondary antibody was added for 1 h at room temperature. Immunoreactive bands were visualized using the Western blotting Luminol reagent, and the images were acquired using an ImageQuant 350 digital image system (GE, USA). Beta-actin (ACTB) was used as a loading reference control.

### Statistical analysis

This study was a descriptive observational study with a blind laboratory analysis for response to treatment. The mean and standard deviation were calculated for the age variable, and the absolute and relative frequency distributions were calculated for the categorical variables. The association between the independent variables and the presence of a clinical response after six months was analyzed by univariate analysis using Pearson’s Chi-square tests or Fisher’s exact tests for categorical variables; SPSS version 21.0 (IBM, USA) was used for statistical analysis. To estimate the independent factors associated with a clinical response at 6 months, all the variables with *p*-values < 0.20 in the univariate analysis were used in the Cox regression. Only variables with *p*-values < 0.05 were retained in the model.

## Results

### Study population

The age of the 78 patients with SCC of the anal canal ranged from 34 to 86 years (mean 63.4 ± 10.3), and 64 patients (82.1%) were female. Analysis of their clinical stage (TNM) showed that the majority of the patients were stage II (60.3%). For the variable T, we found that 30 patients were T2 (38.5%) and 30 were T3 (38.5%). Thus, more than 2/3 (77.0%) of the patients had tumors classified as T2 or T3. Sixty patients (76.9%) clinically evaluated by imaging did not have metastases in their lymph nodes (N0), whereas 18 (23.1%) were positive for lymph node metastases. Analysis of the degree of cellular differentiation showed a predominance of grade II (74.4%). HIV was detected in 18 of the investigated biopsies (23,07%). Nine (64.28%) males and 9 (14.06%) females were HIV positive.

HPV was detected in 59 of the analyzed biopsies (75.6%). HPV 16 was most common and was detected in 49 (62.8%) tumors, followed by HPV 18 in 10 (12.8%) tumors. Nineteen patients (24.4%) did not have detectable HPV infections, and no other type of HPV was found.

Overexpression of p16 was found in 57 patients (73.1%). Overexpression was defined as 50% higher expression of the protein in the tumor tissue than in the normal anal mucosal tissues used as the control. Overexpression was not detected in 19 patients (24.4%) because the amount of protein detected in the tumour was 49% or less compared to the control. Borderline overexpression was found in two patients (2.6%) because protein expression in the tumor was 47% and 50% more than the control.

Analysis of the clinical response at six months showed a complete response in 47 patients (60.3%), and a residual tumor detectable by clinical exam and confirmed by histopathological analysis was found in 31 (39.7%) patients.

### Mutational status and *TP53* copy number and p16 protein expression analyses

*TP53* was wild type in 57 patients (73.1%) and mutated in 21 (26.9%) patients. All cases with p16 overexpression had wild-type *TP53* (73.1%) and vice versa. All the patient characteristics studied are shown in Table 1.

**Table 1:** Patient characteristics

Characteristics	N	%	*HPV Positive* *N (%)*	*HPV Negative* *N (%)*	*p*-value
Age					*0.361*
< 65 years old	44	56.4%			
≥ 65 years old	34	43.6%	*35 (59.3)*	*9 (47.4)*	
Gender			*24 (40.7)*	*10 (52.6)*	**0.004**
Female	64	82.1			
Male	14	17.9	*53 (89.8)*	*11 (57.9)*	
Degree of Cellular Differentiation			*6 (10.2)*	*8 (42.1)*	*0.351*
G I	6	7.7			
G II	58	74.4	*6 (10.2)*	*0 (0)*	
G III	11	14.1	*42 (71.2)*	*16 (84.2)*	
G IV	3	3.8	*8 (13.6)*	*3 (15.8)*	
Clinical Stage (UICC)			*3 (5.1)*	*0 (0)*	**0.031**
I	5	6.4			
II	47	60.3	*5 (8.5)*	*0 (0)*	
IIIA	16	20.5	*39 (66.1)*	*8 (42.1)*	
IIIB	10	12.8	*8 (13.6)*	*8 (42.1)*	
N Stage			*7 (11.9)*	*3 (15.8)*	*0.354*
Negative	60	76.9			
Positive	18	23.1	*47 (79.7)*	*13 (68.4)*	
T Stage			*12 (20.3)*	*6 (31.6)*	**0,012**
T1 (≤ 2 cm)	6	7.7			
T2 (> 2 and ≤5 cm)	30	38.5	*6 (10.2)*	*0 (0)*	
T3 (> 5 cm)	30	38.5	*27 (45.8)*	*3 (15.8)*	
T4 (tumour invading adjacent organ)	12	15.4	*20 (33.9)*	*10 (52.6)*	
HPV			*6 (10.2)*	*6 (31.6)*	*NA*
16	49	62.8			
18	10	12.8	*49 (62.8)*	*0 (0)*	
Negative	19	24.4	*10 (12.8)*	*0 (0)*	
*HIV*			*19 (24.4)*	*0 (0)*	*1.000*
*Positive*	19	24.4			
*Negative*	59	75.6	*14 (23.7)*	*5 (26.3)*	
P16			*45 (76.3)*	*14 (73.7)*	**< 0.001**
Positive	57	73.1			
Negative	19	24.4	*57 (96.6)*	*0 (0)*	
Borderline	2	2.6	*0 (0)*	*19 (100.0)*	
Clinical response at 6 months			*2 (3.4)*	*0 (0)*	**< 0.001**
Yes	47	60.3			
No	31	39.7	*47 (79.7)*	*0 (0)*	
*TP53*			*12 (20.3)*	*19 (100.0)*	**< 0.001**
Mutated	21	26.9			
Wild type	57	73.1	*2 (3.4)*	*19 (100.0)*	
Total	78	100.0	*57 (96.6)*	*0 (0)*	

*NA = Not available*

Statistically significant p-values are shown in bold

**Fig. 1:**
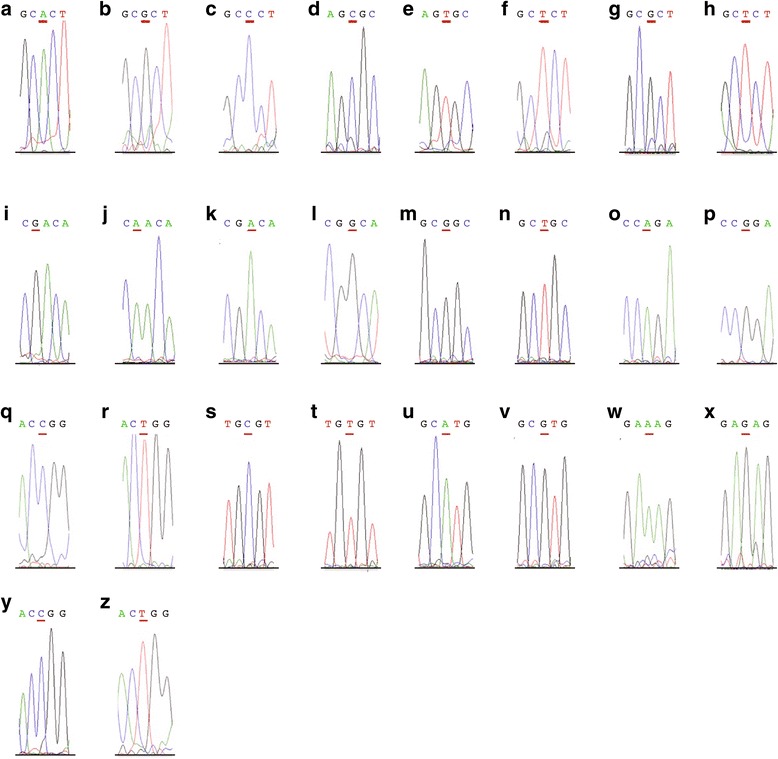
Electropherograms for changes in the *TP53* gene codons (underlined). **a** R175; **b** R175H; **c** R175P; **d** R181; **e** R181C; **f** R181H; **g** R181; **h** R181L; **i** R213; **j** R213G; **k** G245; **l** G245D; **m** G245; **n** G245S; **o** R248; **p** R248Q; **q** R248; **r** 248 W; **s** R273; **t** R273C; **u** R273; **v** R273H; **w** C277; **x** C277F; **y** R282; **z** R282W

Of the 78 patients analyzed, 21 had mutations in exons 5, 6, 7 or 8 of the *TP53* gene. All the mutations identified were homozygous and consisted of both non-functional (80.5%) and partially functional (19.05%) variants (Fig. 1). Because the homozygous state of the mutations may be due to a loss of one allele, cases with mutations in *TP53* were analyzed for copy number by FISH (Table 2).

**Table 2:** Mutational status and copy number analysis for the *TP53* gene and quantification of the centromeres of chromosome 17 in anal canal tumours according to HPV infection status and p16 expression

HPV negative /p16 negative				FISH chromosome 17/ gene *TP53* (%)							
Case	Protein Change	Functional Consequence	Exon	*2/2*	*2/1*	*1/1*	*3/2*	*3/1*	*3/3*	*4/2*	*4/1*
6	R282W	Non-functional	8	60.5	28.0	10.0	1.5	–	–	–	–
7	R181L	Partially functional	5	52.5	43.0	4.5	–	–	–	–	–
11	R273H	Non-functional	8	69.0	23.0	4.5	3.5	–	–	–	–
16	G245S	Non-functional	7	67.5	28.5	2.0	2.0	–	–	–	–
19	R273H	Non-functional	8	70.0	24.5	4.0	1.5	–	–	–	–
23	R175P	Non-functional	5	72.0	20.0	6.0	2.0			–	–
31	R175H	Non-functional	5	67.0	23.0	7.0	3.0	–	–	–	–
34	G245D	Non-functional	7	64.5	20.5	10.0	3.0	2.0	–	–	–
49	R181C	Partially functional	5	48.0	44.0	8.0	–	–	–	–	–
50	R181H	Partially functional	5	44.5	49.5	6.0	–	–	–	–	–
59	R213Q	Non-functional	6	64.0	26.0	6.5	3.5	–	–	–	–
60	R248W	Non-functional	7	76.5	15.0	7.5	0.5	0.5	–	–	–
63	R175H	Non-functional	5	68.0	22.5	5.5	4.0	–	–	–	–
64	C277F	Non-functional	8	63.4	17.0	8.0	4.2	4.2	1.6	1.6	–
70	R273C	Non-functional	8	63.0	19.0	7.5	7.5	2.0	1.0	–	–
72	R248Q	Non-functional	7	65.0	17.5	9.5	4.0	1.5	1.5	1.0	–
75	R248Q	Non-functional	7	67.0	18.5	7.5	3.0	1.5	1.5	1.0	–
76	R213Q	Non-functional	6	65.0	25.5	5.5	4.0	–	–	–	–
77	R273C	Non-functional	8	62.7	24.3	2.7	5.5	2.7	1.6	0.5	–
HPV positive/p16 borderline				*2/2*	*2/1*	*1/1*	*3/2*	*3/1*	*3/3*	*4/2*	*4/1*
29	G245S	Non-functional	7	54.0	24.0	1.5	5.0	5.5	2.5	6.0	1.5
57	R181H	Partially functional	5	59.0	23.0	10.5	1.5	2.0	1.0	1.5	1.5

**Fig. 2:**
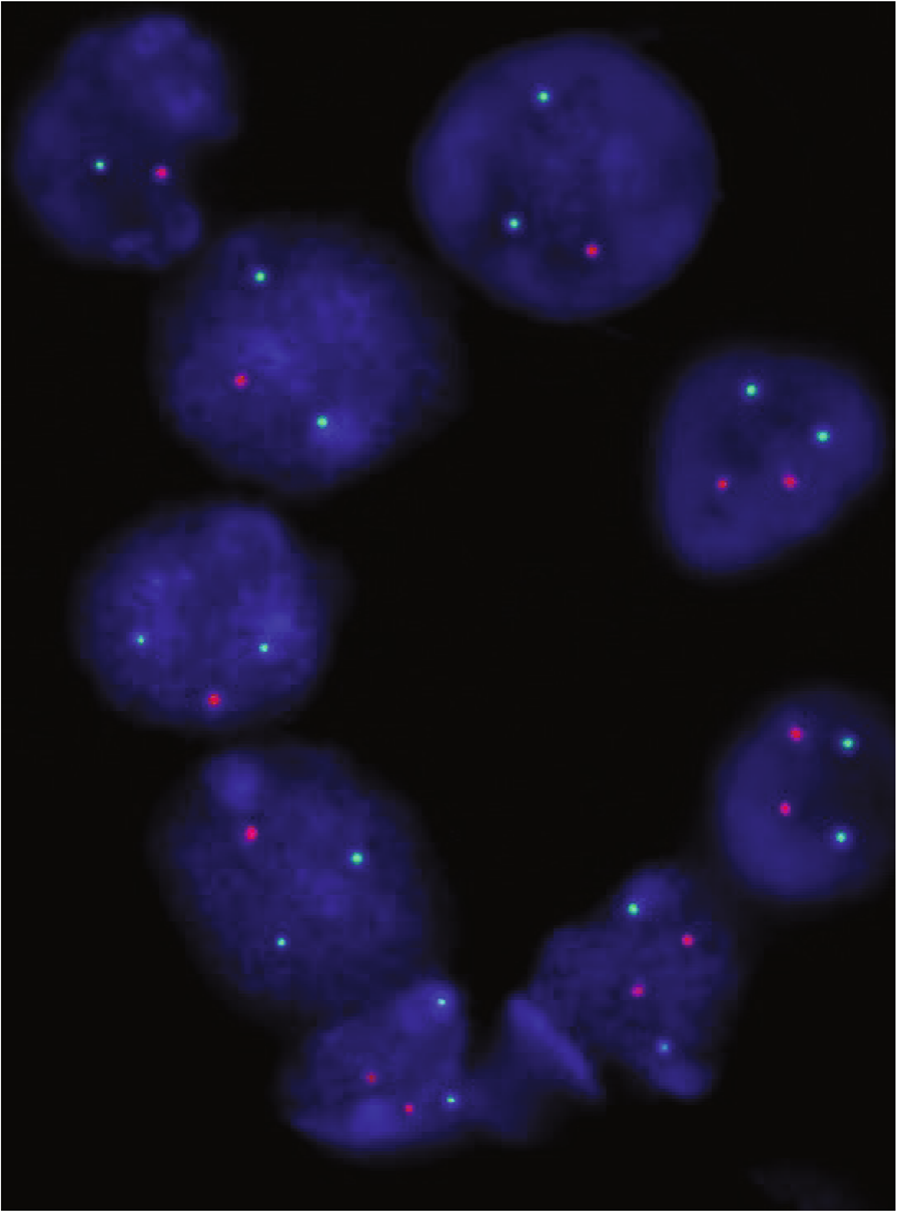
Interphase nuclei from SCC of anal canal cells after FISH analysis. Green points correspond to centromeres of chromosome 17, and red points correspond to alleles of the *TP53* gene

Aneuploidies in chromosome 17, where the *TP53* gene is located, were analyzed to establish a relationship between the number of *TP53* alleles and the number of chromosome 17 copies. Table 2 shows that two chromosome 17 copies and a single allele of the *TP53* gene (2/1) were present between 49.5% and 15% of the cells in cases 50 (Fig. 2) and 60, respectively.

Several other changes were also observed in this analysis (Table 2), including the presence of only one chromosome 17 and one allele of the *TP53* gene (1/1) between 1.5% and 10,5% of the cells in cases 29 and 57, respectively. Additional changes, such as three chromosome 17 copies and two alleles of the *TP53* gene (3/2) and other combinations, were observed in small cellular clones.

Another phenomenon worth highlighting is that 90% of the mutations in the *TP53* gene occurred in tumors that were both HPV negative and did not overexpress the p16 protein, whereas the other 10% occurred in tumors testing positive for HPV and containing borderline overexpression of this protein.

### Factors correlated to the clinical response

**Fig. 3:**
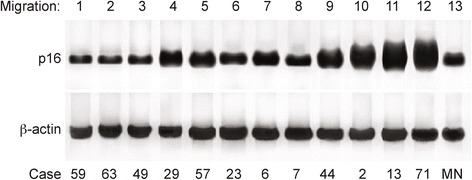
Representative western blot of p16 (upper gel) and beta-actin (lower gel), which was used as an endogenous marker for normalization. Lanes are labelled at the top of the figure, and the case numbers for the patients analyzed are labelled at the bottom. Lanes 1–3 correspond to cases with mutations in *TP53* that are negative for the HPV virus and p16 overexpression. Lanes 4 and 5 correspond to HPV positive cases with mutated *TP53* and borderline p16 overexpression. Lanes 6, 7 and 8 correspond to cases with mutated *TP53* that are negative for HPV and do not overexpress p16 (p16 expression similar to the normal anal mucosa tissue). Lanes 9–12 corresponded to samples with wild-type *TP53* that are positive for HPV and exhibit p16 overexpression. Line 13 corresponds to a pool of p16 proteins extracted from five normal anal mucosal (NM) tissues

The variables with significant associations with the clinical response were HPV, p16, *TP53,* female gender, and stage I or II cancer. Therefore, HPV was highly associated with patients with a complete response to chemotherapy (absence of a residual tumor), with a *p*-value < 0.001. HPV positive tumors showed p16 overexpression 79.7% of the time (*p*-value = 0.003), had borderline overexpression 2.7% of the time and were under expressed 24.4% of the time. Figure 3 shows a representative western blot of p16. The *TP53* gene was wild type in 78.9% (*p*-value = 0.003) of the tumors positive for HPV (Tables 3 and 4).

**Table 3:** Presence of a clinical response at 6 months by selected factors

Characteristics	Clinical response (%)	No clinical response (%)	*p*-value
Age			*0.488*
< 65 years old	28 (63.6)	16 (36.4)	
≥ 65 years old	19 (55.9)	15 (44.1)	
Gender			**< 0.001**
Female	46 (71.9)	18 (28.1)	
Male	1 (7.1)	13 (92.9)	
Degree of Cell Differentiation			*0.387*
G I / G II	40 (62.5)	24 (37.5)	
G III / G IV	7 (50.0)	7 (50.0)	
Clinical Stage (UICC)			**< 0.001**
I - II	31 (86.1)	5 (13.9)	
III A - III B	16 (38.1)	26 (61.9)	
T stage			**< 0.001**
T 1 (≤2 cm)	1 (16.7)	5 (83.3)	
T 2 (> 2 and ≤5 cm)	4 (13.3)	26 (86.7)	
T 3 (> 5 cm)	15 (50.0)	15 (50.0)	
T 4 (tumour invading adjacent organ)	11 (91.7)	1 (8.3)	
N stage			**0.035**
Negative	40 (66.7)	20 (33.3)	
Positive	7 (38.9)	11 (61.1)	
HPV			**< 0.001**
Positive	47 (79.7)	12 (20.3)	
Negative	0 (0.0)	19 (100.0)	
HIV			*0.063*
Positive	8 (42.1)	11 (57.9)	
Negative	39 (66.1)	20 (33.9)	
P16			**< 0.001**
Positive	45 (78.9)	12 (21.1)	
Negative/borderline	2 (9.5)	19 (90.5)	
*TP53*			**< 0.001**
Mutant	2 (9.5)	19 (90.5)	
Wild type	45 (78.9)	12 (21.1)	

Statistically significant *p*-values are shown in bold

**Table 4:** Univariate and multiple analyses of factors associated with the clinical response at 6 months

Characteristics	OR	95% CI	*p*	aOR	95% CI	*p*
HPV positive	31.1	1.8–531.1	**0.018**	31.1	1.8–531.1	**0.018**
Female gender	10.1	1.4–73.0	**0.022**			
P16 positive	8.3	2.0–34.2	**0.003**			
Age < 65 years	1.1	0.6–2.0	0.662			
Degree of cell differentiation I and II	1.3	0.6–2.5	0.586			
Clinical stage I and II	2.5	1.3–5.0	**0.008**			
T 2/3	1.2	0.5–3.1	0.675			
*TP53* mutated	0.1	0.03–0.5	**0.003**			

OR = Odds ratio; aOR = Adjusted odds ratio

Statistically significant *p*-values are shown in bold

After adjusting for variables with a *p*-value < 0.20 in the univariate analysis (Table 4), only the presence of HPV was independently correlated with the clinical response. This variable increased the chances of a response within six months by 31-fold.

## Discussion

Tumors affecting the anal canal generally regress slowly after chemoradiotherapy [21]. Favorable clinical outcome at three months seems to be more important than the initial T and N stages, and the magnitude of tumor regression (over 80%) is a better predictor of both colostomy and disease-free survival [22]. In our study, we observed that the female gender (*p* = 0.022) and clinical stages I and II (*p* = 0.008) were predictive variables for a good local response and significantly increased the chances of responding to treatment compared to males and the more advanced stages III A and III B. In our analysis, tumor size and lymph node metastases were not identified as independent predictive variables.

The evaluation of complete response after six months of chemoradiotherapy and the correlation with the biomarkers analyzed in this study is a significant parameter, but it is not definitive. A 3-year locoregional control rate would be considered the most relevant evaluation because it provides information on recurrences (local and distant), which occur predominantly in the first year after treatment [23]. We did not follow-up for three years because most of the patients who participated in this study did not return to the hospital after the six-month visit.

HPV status is recognized as prognostic factor in head and neck carcinomas, where it is agreed that positive HPV patients react well to chemoradiotherapy. Interest in identifying the role of HPV in SCC of the anal canal has increased because patients with HPV positive tumors in the head and neck region, especially the oropharyngeal region, have better overall survival. Thus, the role of high risk HPV in the aetiology of SCC of the anal canal is well established [4, 5, 21, 24–29].

A complete clinical response, i.e. the absence of residual tumour after six months of the end of treatment with chemoradiation, is considered an excellent predictor of local control. In fact, several studies have shown that after this period, an 80% regression in T3 and T4 tumors is predictive of disease-free survival in two years: 66% versus 20% for responders and non-responders, respectively [22, 28].

In this study, the presence of HPV increased the chance of absence of residual tumors, within six months, by 31-fold. HPV was detected in 75.6% of the biopsies; of these, 79.7% (*p* = 0.018) showed complete local regression, and 20.3% had residual tumors after six months. However, all the patients with HPV negative biopsies (24.4%) had residual tumors, which is higher than expected when compared to data published in international studies [6, 7, 10, 11, 30]. All patients who had detectable residual lesions underwent a CT of the chest and MRI of their abdomen and pelvis to analyze the distant metastasis and regional lymph node chains.

In a retrospective study, Yhim et al. found similar results. They analyzed the prognostic significance of HPV type 16 after an oncological follow-up of 51.7 months in patients with SCC of the anal canal treated with chemoradiation and found a disease progression-free period in 63.1% of the HPV positive patients compared to 15.6% of the HPV-negative patients and an overall survival of 84.6% versus 39.8%, respectively [6].

Other studies have also analyzed HPV and p16 expression as markers of the prognostic outcome in patients with SCC of the anal canal and corroborated the results found in this study. Koerber et al. analyzed the HPV and p16 statuses in 105 patients with SCC of the anal canal in a study cohort. The average follow-up was 48.6 months, and the analysis showed that the overall survival and progression-free times were three years longer in the HPV positive patients than in the HPV-negative patients. The authors also concluded that HPV detection and p16 expression together represented a prognostic marker in patients with SCC of the anal canal [8]. Mai et al. conducted a study that analyzed the prognostic relevance of HPV combined with p16 expression and observed that locoregional clinical control was significantly higher in stage T1/T2, female, HPV positive, and p16-overexpressing patients [9].

Meulendijks et al. performed a cohort study in 107 patients with SCC of the anal canal undergoing standard treatments. They demonstrated that the HPV and p16 statuses were strong predictors of locoregional clinical control and overall survival. The outcomes of patients with HPV negative/non-p16-overexpressing tumors were considerably worse than those of patients with HPV positive/p16-overexpressing tumors. Similar to our study, they found a high frequency of p53 mutations in the HPV negative/non-p16-overexpressing tumors (80%) and a sporadic frequency in the HPV positive/p16-overexpressing tumors (6%) [10]. However, unlike our results, they observed that 4 of the 16 HPV negative tumors had p16 overexpression (25%). Similarly, in a population of 135 patients, Serup-Hansen et al. found that 6 out of 15 HPV negative tumors (40%) showed overexpression of the p16 protein [30].

*TP53* sequence analysis of the 78 tumor samples showed that 21 patients had tumors with homozygous mutations in this gene (Table 2). Therefore, the copy number analysis by FISH was performed on *TP53* mutations to verify whether the mechanism of the loss of heterozygosity (LOH) was involved in the carcinogenicity of the anus, i.e. to confirm whether the mutation is homozygous. This was visualized by the Sanger sequencing method, to determine if it originated from the two mutated alleles or because only one allele was sequenced since the other allele was lost by deletion and gave the false impression of homozygous [31, 32].

The FISH analysis (Table 2) revealed the presence of two chromosome 17 copies and a single allele of the *TP53* gene (2/1) in 15% to 49.5% of the analyzed tumor cells, indicating that LOH is a mechanism for the activation of this tumour suppressor gene. Another population of cells with homozygous mutations in the *TP53* gene that could be explained by a similar mechanism was the clone with only one chromosome 17 and one allele of the *TP53* gene (1/1) that was found in 1.5% to 10.5% of the cells in the analyzed samples.

Additionally, the typical diploid ratio of normal somatic human cells was also observed in 44.5% to 76.5% of the cells in the 21 analyzed cases. For these cellular clones, which represent most of the cells in the tumor, there are two other mechanisms to explain the homozygosity of the mutations. The first explanation is the occurrence of sporadic mutations in the two alleles of the tumor cells, and the second is gene conversion [33].

Other changes, such as the presence of three chromosome 17 copies and two *TP53* alleles (3/2) and other combinations, were observed in small clones of cells. These homozygous mutations can also be explained by the two mechanisms cited above.

Another significant phenomenon is that 100% of the HPV negative tumours had mutations in the *TP53* gene. This change in the genome of the anal mucosal cells could cause the formation of the tumor because the loss of p53 suppression might be directly related to carcinogenesis in some types of cancer [34].

*TP53* mutations may explain the process of carcinogenesis in the anal canal in 3.4% of the HPV positive tumors. Additionally, the mechanism of carcinogenesis might be related to the dysregulation of *TP53* by the viral oncogene E6 in the 96.6% of the tumors that were HPV positive but did not have mutations in this gene [35].

The SCC model of the anal canal may also explain why the presence of the virus gives a better response to therapy across the spectrum of HPV associated tumors (oropharynx, cervix, vulva, penis) [2]. The presence of *TP53* mutations is associated with worse outcomes after chemoradiotherapy and is inversely correlated with the presence of p16 / HPV [7]. Mutations of *TP53* and HPV involvement are, for the most part, mutually exclusive in SCC of the anal canal [10] and in head and neck cancer [36]. HPV positive tumors develop through the production of the E6 oncoprotein and chemoradiotherapy can disrupt this process causing significant DNA damage. In the absence of E6 (i.e., HPV negative cases), the *TP53* mutation is irreversible and the DNA-damage-response (DDR) mechanisms are no longer orchestrated by the p53 protein, which can cause increasingly aberrant cells after treatment [2, 37].

The difference between these two mechanisms to inactivate the activity of the p53 protein may explain why HPV negative patients generally do not respond to treatment within 6 months. Our hypothesis proposes that the mutations in *TP53* found in the tumor cells of HPV negative patients produce abnormal proteins that confer chemoresistance to the administered treatment. Conversely, the majority of HPV positive tumors (79.7%) responded well to chemotherapy, which is explained under our hypothesis by their production of wild-type p53; even though p53 was degraded by the viral E6 oncoprotein, other proteins were concurrently generated to maintain a basal level of the protein that likely prevented the onset of chemoresistance.

Patients with SCC anal canal tumors with mutations of *TP53* gene are associated with short-term disease-free survival and decreased local responses [3, 38–42]. These observations are consistent with our results, where a significant majority of the mutant *TP53* tumors did not show a local response, although we did not analyze disease-free survival.

Individuals infected with HIV are at increased risk of infection with both low-risk and high-risk HPV types. Chronic immunosuppression provides an environment for persistent HPV infection which carries a higher risk of malignant transformation [43]. In this study, HIV infection was not associated with the presence of HPV and/or clinical response in SCC, reinforcing the hypothesis that HPV infection is only one of the mechanisms of anal canal carcinogenesis.

The explanation for the favorable clinical outcome of chemoradiotherapy in HPV16 positive patients is evident in tumors harboring strong infiltration of immune cells. These tumors present tumor-infiltrating lymphocytes (TILs) with a strong expression of CD8 and the immunological markers Programmed Cell Death Protein-1 (PD-1) and its ligand (PD-L1) [44, 45] Patients with SSC of the anal canal with high levels of infiltration of TILs present a relapse-free rate higher than those with low levels of infiltration of TILs [46]. For this reason it is important that the immune recognition in SSC of the anal canal is measured systemically.

## Conclusions

Only the presence of HPV increased the chance of absence of residual tumors, within six months, by 31-fold. This correlation is valuable and can direct future therapeutic approaches in SCC of the anal canal.

## Abbreviations

ACTB: Beta-actin
CMT: Combined Modality Therapy
CT: Computer Tomography
FDG: 2-Deoxy-2-[18F] fluoro-D-glucose
FFPE: Formalin-Fixed Paraffin-Embedded
FISH: Fluorescent In Situ Hybridization
HIV: Human Immunodeficiency Virus
HOL: Hospital Ophir Loyola
HPV: Human Papillomavirus
MRI: Magnetic Resonance Imaging
NM: Normal Anal Mucosal
PCR: Polymerase Chain Reaction
PET/CT: Positron Emission Tomography–Computed Tomography
PVDF: Polyvinylidene Fluoride
SCC: Squamous Cell Carcinoma
TNM: Cancer Tumour–Node–Metastasis
